# Non-Typhoidal *Salmonella* Infection in Children: Influence of Antibiotic Therapy on Postconvalescent Excretion and Clinical Course—A Systematic Review

**DOI:** 10.3390/antibiotics10101187

**Published:** 2021-09-29

**Authors:** Johanna L. Leinert, Stefan Weichert, Alexander J. Jordan, Rüdiger Adam

**Affiliations:** University Children’s Hospital, Medical Faculty Mannheim, Heidelberg University, 68167 Mannheim, Germany; johanna.leinert@umm.de (J.L.L.); stefan.weichert@umm.de (S.W.); alexander.jordan@umm.de (A.J.J.)

**Keywords:** non-thyphoidal *Salmonella*, antibiotic treatment, fecal excretion, children

## Abstract

(1) Background: Although published recommendations are available, the use of antibiotics in non-typhoidal *Salmonella* (NTS) infections in children is still controversially debated in clinical practice. Patients might even be put at risk, with necessary antibiotic therapy being withheld due to the widespread concern of prolonged post-convalescent shedding. The authors conducted a systematic review to assess whether antibiotic treatment influences fecal excretion or the clinical course in children with NTS infection. (2) Methods: The review was carried out following the PRISMA guidelines. In a Medline database search, studies assessing the influence of antibiotic therapy on excretion and/or the clinical course of NTS infections were selected. Studies reporting on adults only were not considered. Out of 532 publications which were identified during the systematic literature search, 14 publications were finally included (3273 patients in total). Quality and bias assessment was performed using the Newcastle-Ottawa scale (NOS) or the Cochrane risk-of bias tool (ROB-2). (3) Results: Four early studies from decades ago demonstrated a prolongation of intestinal NTS excretion in children after antibiotic treatment, whereas most studies published more recently observed no significant influence, which might be due to having used more “modern” antibiotic regimes (*n* = 7 studies). Most studies did not describe significant differences regarding the severity and duration of symptoms between untreated patients and those treated with antibiotics. Quality and bias were mainly moderate (NOS) or variable (ROB-2), respectively. (4) Conclusions: There is no substantial evidence of prolonged excretion of NTS in pediatric patients after treatment with newer antimicrobials. Consequently, clinicians should not withhold antibiotics in NTS infection for children at risk, such as for very young children, children with comorbidities, and those with suspected invasive disease due to concerns about prolonged post-convalescent bacterial excretion. In the majority of cases with uncomplicated NTS diarrhea, clinicians should refrain from applying antibiotics.

## 1. Introduction

*Salmonellae* are gram-negative facultative intracellular enterobacteria. As the taxonomic classification is somewhat intricate in daily practice, clinicians still subdivide the anthropopathogenic serovars of *Salmonella enterica* subspecies I *enterica* into typhoidal and non-typhoidal *Salmonellae* (NTS), with *S. typhimurium* and *S. enteritidis* being the most prominent non-typhoidal serotypes. NTS are one of the most common bacterial pathogens worldwide causing enterocolitis, with an estimated 95 million cases globally in 2017 being a burden both in developing and developed countries [1].

NTS are present in a wide range of reservoirs and hosts, mainly in poultry, and less frequently in other animals, like reptiles or amphibians kept in households, as well as in humans themselves. Infections with NTS are usually associated with contaminated food [2], most commonly chicken and eggs, but also many other food products such as meats, nuts, spices or produce [3]. Apart from foodborne NTS acquisition, animal contact, ingestion of contaminated water, as well as person-to-person transmission may be sources of infection [4,5]. Direct horizontal spreading between humans might be even more important in developing countries than in other regions of the world [6,7]. It is believed that there is a high number of undetected NTS infections; one estimate suggests 29 infections for every culture-confirmed case [8].

The most commonly encountered noninvasive NTS-disease is clinically hard to distinguish from enteric infections caused by other microbes. It usually presents as self-limiting diarrhea, abdominal pain and, to a lesser extent, nausea, vomiting, or fever [9,10]. In most of the cases these symptoms are mild or the NTS infection might even be asymptomatic.

In parts of the developing world, though, notably sub-Saharan Africa, NTS have even become a predominant invasive pathogen following the effective establishment of vaccination programs against *Streptococcus pneumoniae* and *Haemophilus influenzae* type b. Sometimes patients present “typhoid-like” or with the picture of enterocolitis, high fever, and an acutely ill appearance. Rarely, such patients might even be misdiagnosed with NTS infections in cases of perforated appendicitis with diarrhea [11]. However, invasive disease *per se* only presents with diarrhea in roughly one third of the patients [12,13]. It typically manifests as bacteremia, either alone or with subsequent seeding resulting in endocarditis, pneumonia, or focal suppurative infections, such as visceral abscesses, osteomyelitis, or arthritis [14,15,16,17]. Immunodeficiencies, infections with human immunodeficiency virus (HIV) or malaria, sickle cell disease, malignancies, malnourishment, or medication with immunomodulatory drugs (e.g., corticosteroids) predispose children for invasive disease and consequently for complications [18,19]. This risk is even more pronounced in younger children and particularly in infants [20]. Especially in such young patients, the most feared complication of NTS infection is the development of a meningitis, which can lead to severe neurological damage and is associated with a high rate of mortality [21,22].

Principally, prolonged carriage or fecal shedding is a well-recognized consequence of intestinal NTS infection, especially in young children under the age of five [23,24]. In some instances, such periods can extend to months or rarely even years [25]. In a meta-analysis that included 486 children younger than five years, 2.6% of the patients excreted *Salmonellae* for one year or longer [26]. Apart from convalescent pediatric patients, asymptomatic children without an obvious history of diarrheal illness may also be a considerable source for *Salmonella* spreading. In an unselected pediatric population without diarrheal symptoms, the carriage rate was found to be 1% in more than a thousand schoolchildren in India [27]. Therefore, especially in low-income regions, NTS-invasive disease remains a significant burden of disease resulting in relevant morbidity and mortality [1,28,29,30], and identification of the patients needing adequate antibiotic therapy is of great importance.

The use of antibiotics for the treatment of such NTS infections was broadly discussed in the last decades, while the antibiotic regimens applied have undergone substantial modifications during this time [23,31,32,33]. This development was at least in part driven by changes in antimicrobial resistance patterns of *Salmonella* spp. and acknowledgement of the importance of more favorable pharmacokinetic properties of the antimicrobial agents applied [34,35]. Throughout the years, though, the impact of antibiotic therapy on the clinical course of the infection, on the duration of symptoms, and on the time of post-convalescent intestinal excretion of NTS has remained a matter of debate [36,37,38].

Accordingly, the objective of this work was to shed light on the value of antibiotic therapy for NTS infection in children focusing on the impact on intestinal shedding and on the clinical course of the disease based on the current literature.

## 2. Materials and Methods

The methodology of our review was consistent with the Preferred Recording Items for Systematic Reviews and Meta-Analyses (PRISMA) recommendations. The corresponding checklist is provided in Table S1 (available as Supplementary Data).

A review of the literature was performed independently by two investigators (J.L.L. and R.A.) using the MEDLINE database. The search algorithm was constructed using the following medical subject heading terms (MeSH) and text words in various combinations: “*Salmonella*”, “Salmonellosis”, “infections”, “enteritis”, “enterocolitis”, “food poisoning”, “nontyphoid”, “non-typhoidal *Salmonella*”, “NTS”, “bacteremia”, “children”, “childhood”, “infants”, “pediatric”, “infancy”, “antibiotics”, “antibiotic therapy”, “antimicrobial therapy”, “fecal excretion”, “shedding”, “post-convalescent”, “excretion”, “symptoms”, and “clinical course”.

The search was not restricted by the date of publication or the type of studies. After the literature search, titles and abstracts were screened to identify potentially appropriate articles. Subsequently, a detailed evaluation of these articles including crosschecking of the references was performed. Only studies reporting on children and adults or children only were included. Studies about adults only were excluded from this review. Only original articles (no reviews or case reports) were included in this analysis. Data acquisition started in October 2020 and was finished in July 2021.

The assessed characteristics of the considered articles included the type of study (e.g., randomized controlled trial or retrospective case series), the pathogen, the number of patients, the ratio of children (<18 years), and the administered antibiotics. The findings of these studies were investigated with the focus on the influence of antibiotic treatment on the intestinal excretion of NTS or on the clinical course of patients, respectively.

Quality assessment was performed independently by two investigators (J.L.L. and R.A.). Any disagreement in scoring was solved by general consensus.

For non-randomized studies, the Newcastle-Ottawa scale [39] was used (Table S2); for randomized-controlled trials, the Cochrane risk-of bias tool for randomized trials (ROB-2) [40] was applied.

## 3. Results

**Figure 1:**
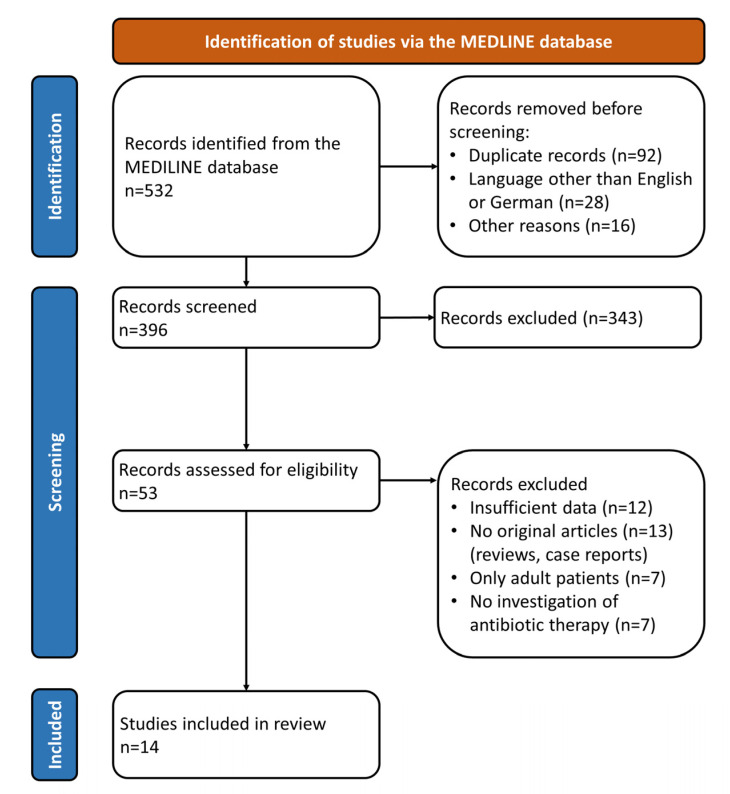
Illustration of the study selection process.

A flow-chart illustrating the study selection is shown in Figure 1. The systematic literature search identified 532 records, of which 396 scientific articles were screened for inclusion in this review. After screening of titles and abstracts, 343 articles were excluded because of irrelevance or unsuitability according to the abovementioned selection criteria. Hereafter, 53 potentially appropriate articles were evaluated in detail. Furthermore, 39 articles were excluded for the following reasons: insufficient data, no original articles (reviews or case reports), only adult patients, and/or no systematic consideration of antibiotic therapy. A total of 14 scientific articles published between 1954 and 2021 were subsequently assessed for this review [13,23,31,32,37,41,42,43,44,45,46,47,48,49].

The results of the quality assessment are described in Tables S3 and S4. Of the non-randomized trials, eight studies were rated “moderate quality” and one study “low quality”. Of the randomized-controlled trials, the overall risk of bias was “low” for two studies and “high” for three studies.

Altogether, the selected studies comprised 3273 patients, with 95.8% of them being children (12 studies included children only, while in two studies, the rates of children were 95.1% and 64.3%, respectively). An overview of these articles is shown in Table 1.

Of these studies, five were prospective randomized controlled trials, five were retrospective case series, two were prospective controlled trials, and two were prospective case series. The randomized controlled trials were allocated between “antibiotic treatment” and “no antibiotic treatment” or placebo in patients with NTS infection [41,42,43,45]. In one of the prospective controlled trials, patients with severe enterocolitis were assigned to antibiotic treatment, while the rest received supportive treatment only [48]. A recent prospective case series compared children with appropriate, inappropriate, and no antibiotic treatment [47]. The other articles with an observational study design investigated the influence of antibiotics on the duration of NTS excretion without a randomization between antibiotic treatment and a control group.

The sub-group of *Salmonella* spp. was not specified in all of the investigated studies. In 12 of 14 studies, all NTS species were included, whereas in two studies *S. typhimurium* was the predominantly diagnosed pathogen. The antibiotic agents which were used changed over time. The most commonly administered substances before 1990 were chloramphenicol, ampicillin, and neomycin, while in more recent studies, fluoroquinolones and cephalosporins were predominantly applied.

In 13 articles, the duration of excretion was investigated [23,31,32,37,41,42,43,44,45,47,48,49]. Prolongation of NTS shedding after antibiotic therapy was found in four studies (including 127, 117, 70, and 239 patients, respectively) [32,37,41,49]. In 1965, Dixon et al. published an analysis of two similar outbreaks in schools after food poisoning caused by *S. typhimurium* [32]. Seven weeks after the outbreak, the percentage of positive fecal samples was 44.8% for children with antibiotic treatment versus only 4.7% for untreated children. Accordingly, two years later, Rosenstein et al. reported on an investigation of *Salmonella* excretion of 70 patients with symptomatic and asymptomatic NTS infections and found that patients who were treated with antibiotics had a substantially longer excretion time [37]. After twelve weeks, stool cultures were negative for all patients who did not receive antimicrobial treatment compared with 30.1% positive stool cultures in those patients who were treated.

These results were confirmed by a further study that demonstrated that patients who were given antibiotics were more likely to be symptomless excreters in a six-week period after treatment than those given a placebo (lactose) [41].

Ho et al. also reported a prolongation of fecal shedding, albeit only in children receiving inappropriate antibiotic treatment defined as either not active in vitro or given to patients without severe disease. They did not observe a prolongation of intestinal NTS excretion after adequate antibiotic therapy (active in vitro or given to patients with a high severity score) or in the group receiving no antibiotics [47].

Shortening of the duration of excretion was found in one study (including 30 patients) published by Chiu et al., who observed a significantly higher rate of clearance of NTS from stools after antibiotic therapy using ceftriaxone in children with bacteremia [48]. The authors compared two groups of 15 patients with NTS enterocolitis and bacteremia receiving treatment with ceftriaxone or no treatment, respectively. One month after therapy, the rate of NTS excretion was 20% for patients treated with ceftriaxone versus 63% for those without antibiotic treatment.

No influence of antibiotics on NTS excretion was found in six studies (including, in total, 1865 patients) [23,31,42,43,44,45]. In the largest study focusing on this topic, comprising 1543 patients, of which 21.2% had received antibiotics during an acute NTS infection for a mean duration of six days, Barbara et al. reported that antibiotic treatment did not affect the fecal shedding at any time point up to three months post-infection as compared with age-matched untreated controls [23].

All four studies that observed a prolongation of NTS excretion after antibiotic treatment in children were published in 1974 or earlier. No such phenomenon was reported since then in the literature assessed for this review. 

The impact of antibiotic treatment on the clinical course of the infection was reported in 11 studies [13,23,31,41,42,43,45,46,47,48,49]. Six out of these studies did not observe a significant influence of antibiotic treatment on the severity and/or duration of symptoms [31,41,42,43,45,48]. The first of these studies was published by Macdonald et al. in 1954 [31]. In this early study, comprising 51 children, the mean duration from the beginning to clinical cure was similar: 19.5 days for treated children and 19.0 for the control group. The largest of these studies (168 patients, 64% children <12 years) that assessed the duration of symptoms also did not observe an overall significant difference between patients treated with neomycin and those treated with placebo [41]. Most of the works which did not observe a clear influence of antibiotic treatment on the clinical course were performed in earlier years.

In one study, a higher frequency of persistent symptoms after antibiotic therapy was observed [23]. In this investigation, performed three months after NTS infection, 6.0% of the subjects still complained of digestive symptoms, such as vomiting, abdominal pain, and diarrhea. The rate of patients reporting such symptoms was 9.5% in the group that received antibiotic therapy, compared with 2.9% in those who did not receive it.

A recent study observed a worse clinical outcome only in children with inappropriate antibiotic treatment, but not after treatment with appropriate antibiotic agents [47]. The authors stratified 126 children into “no antibiotic therapy” (*n* = 69 patients), “appropriate antibiotic therapy” (bacteremia or severe cases receiving antibiotics active in vitro; *n* = 24 patients), and “inappropriate antibiotic therapy” (cases with mild or moderate severity receiving antibiotics resistant in in vitro testing; *n* = 33 patients) groups. The “appropriate antibiotic therapy” group had a similar fecal excretion time compared to the “no antibiotic therapy” group (9.64 ± 6.22 days versus 11.13 ± 7.01 days). In a multivariate analysis, the authors showed that an inappropriate antibiotic therapy significantly prolonged the fecal excretion time (18.24 ± 24.67 days).

Further subsequent works on this subject, such as the ones published by Lin et al. in 2003 and by Tsai et al. in 2011, reported potential positive effects of a short-term antibiotic therapy for three to five days [13,46]. Lin et al. analyzed patients with a severe clinical cause which was defined as high C-reactive protein (CRP) levels, high fever, and signs of dehydration, and showed that a therapy using short-term ceftriaxone was clinically beneficial for this group of patients (e.g., rapid defervescence after 1.6 ± 1.4 days (mean ± standard deviation)). Based on a retrospective analysis of 683 children (386 with antibiotic treatment and 297 without), Tsai et al. similarly concluded that critically ill patients with high CRP levels and longer febrile duration prior to admission might benefit from the administration of third generation cephalosporins or fluoroquinolones. Treatment with these antibiotic agents resulted in a shortened duration of fever and in fewer days of hospitalization. Table 2 summarizes the findings of this systematic review.

## 4. Discussion

The adequate therapy for children suffering from infections caused by NTS species is principally well described in published recommendations [50,51,52,53,54]. International guidelines are scarce, though, and rarely address invasive NTS infections specifically [55]. The risk of prolonged excretion of *Salmonella* in pediatric patients treated with antibiotics is still a relevant matter of debate in routine clinical practice. Substantial insecurity about this issue among colleagues is not rare (as perceived in cases admitted to our university children’s clinic as a tertiary referral hospital specialized in infectious diseases). Warnings to varying extend about this assumed risk can be found in virtually all publications on the subject of NTS infections in children. In addition to the aspect of prolonged shedding, a second issue frequently discussed is the effect of antibiotic therapy on the severity and duration of symptoms in pediatric patients with invasive NTS infection.

The uncertainty regarding the administration of antibiotics is based on several clinical studies published over the last decades which reported both positive and negative, but also often nonsignificant effects of an antibiotic treatment for NTS infection on *Salmonella* excretion and on the patients’ symptoms [13,23,31,32,36,37,41,42,43,44,45,46,47,48,49,56,57,58,59].

The very first study that systemically assessed these factors was the work by MacDonald et al. published in 1954 [31]. The authors observed neither a prolongation of NTS excretion nor an influence of antibiotic therapy on the clinical course. The first study which reported a prolongation of fecal shedding after antibiotic therapy was published by Dixon et al. in 1965 [32]. However, the cohorts described were most likely infected by different NTS strains, as the two outbreaks examined occurred ten years apart and in different geographical regions and the antibiotic regimens used were highly diverse, with one to three courses of various antibiotic agents applied. Furthermore, as almost all of the affected school children received antimicrobial medication, it is likely that students not severely affected were treated as well. In consequence, the conclusion of the study of a possible prolongation of fecal NTS shedding after antimicrobial therapy has to be perceived with caution.

Another study describing protracted clearance of NTS after antibiotic treatment was published by Rosenstein et al. in 1967 [37]. Limitations of this study include different age spectra of the untreated and the treated group (e.g., 68% vs. 10% in the age group 15 years or older), detection of NTS infection by a single culture, often from a rectal swab, and antibiotic treatment of relevant numbers of asymptomatic patients.

Further work propagating a lower clearance of NTS in antibiotically treated patients was published by the “Joint Project by Members of the Association for the Study of Infectious Disease” in 1970 in a randomized controlled trial, in which 168 patients (108 < 12 years) were assigned to receive either oral neomycin or placebo (lactose). Clearance rates and differences in fecal NTS excretion for the whole cohort including adults were calculated after just three weeks. For a fraction of 76 patients, there was a tendency for prolonged shedding after six weeks following neomycin treatment (36% vs. 25%). No clear differentiation between age groups was performed and severity of symptoms was not assessed. Therefore, the conclusions drawn by the authors regarding the influence of antibiotics on NTS shedding is debatable.

One more study described a prolongation of NTS excretion after antibiotic treatment in 117 children. Here, the tendency of protracted *Salmonella* clearance was discrete and the authors themselves stated that this effect may not have been related to antibiotic therapy alone, as it was applied most frequently in infants less than 3 months of age and mainly in very sick patients [49].

All studies that reported a prolongation of fecal NTS excretion in pediatric patients were published no later than 1974. Since then, only one observational study, which predominantly included adult patients, also observed a prolongation of excretion. In this observation, 51 out of 105 patients were followed with stool cultures subsequently to a foodborne *S. typhimurium* outbreak. Fifteen children seven years or older were included, eight hospitalized and antibiotically treated, of whom three children excreted NTS longer than four weeks. It remains unclear whether disease severity itself led to hospitalization or the antibiotic therapy *per se* was responsible for prolonged shedding in the small number of children [36]. Although frequently cited in the literature in support of the notion that administration of antimicrobial drugs extends NTS excretion, such a conclusion should be appreciated with caution, as only a fraction of the original cohort was followed and the number of children was small.

Inappropriate use of antibiotics, however, does seem to significantly prolong the time of excretion, as shown in patients with mild disease receiving antibiotics or patients with severe disease receiving antibiotics later being found to be resistant in vitro [47,60]. So, in accordance to published guidelines and generally accepted assumption, these results confirm that, especially for mild clinical courses of NTS diarrhea, empiric antibiotic treatment should generally be avoided [33,38,52].

Another parameter identified as a risk factor for a prolonged excretion was an antibiotic therapy course longer than seven days, suggesting that a short-term antibiotic treatment might be beneficial [61]. The adequate duration of the treatment has not yet been systematically investigated in larger clinical studies, though.

The majority of the studies assessed which were published in the last decades, including three randomized controlled trials, could not demonstrate any significant influence of antibiotic therapy on the duration of excretion [23,42,43,45]. One prospective controlled trial even observed a significantly shorter duration of NTS excretion after antibiotic treatment using ceftriaxone [48].

When weighing the possible risk of bacterial shedding and the potential influences of an antibiotic therapy, it also has to be considered that even without antibiotic treatment, the excretion of NTS is often prolonged, lasting up to 20 weeks especially in young children (under the age of five) [62].

A possible explanation for the higher rate of prolonged fecal excretion in patients treated with antibiotics can be derived from the design of most of the non-randomized studies. In many studies, the indication for an antibiotic treatment was mainly based on the severity of the disease and the patients’ age. Consequently, especially very sick and young children had received antibiotics. Therefore, one possible explanation for the observed prolongation of bacterial shedding could be a presumably higher bacterial load in these more severely affected and young children leading to higher concentrations of NTS in the stool independent from an antibiotic intervention.

Apart from the impact of antibiotic treatment on fecal excretion, the influence on patients’ symptoms and the clinical course of the disease might be even more important, especially for patients at risk, such as very young children as well as immunocompromized patients and those with co-morbidities. The majority of the analyzed studies reported no significant difference regarding the severity or duration of the children’s symptoms after antibiotic therapy. The most recent studies even reported potential benefits of an antibiotic treatment using ceftriaxone for a subgroup of patients with a severe clinical course [13,46,63]. However, clinical symptoms of the patients that were included in this review were relatively heterogeneous, ranging from asymptomatic courses to severe disease, which was mainly defined by fever, CRP levels and/or bacteremia, as well as the need for hospitalization and the severity of diarrhea.

As mentioned above, the armamentarium of antibiotic agents has broadened in the last decades, with regimes being refined regarding the duration of treatment and the substances used. Chloramphenicol, for example, is nowadays considered as reserve antibiotic and is even not officially approved for young children in some parts of the world. “Newer” antibiotic agents, such as third-generation cephalosporins, did not cause a prolongation of excretion and were associated with potentially beneficial effects on the patients’ symptoms [46,48]. Adequately powered clinical studies are warranted to evaluate the efficacy of these drugs for patients with NTS infections.

Another promising group of antibiotics for the treatment of NTS infection are fluoroquinolones [63,64]. However, the studies that investigated these agents included adult patients only. Fluoroquinolones should be prescribed with caution in children because of potential side effects, albeit their application should be considered in life-threatening instances involving the central nervous system, such as NTS-meningitis, because of their favorable blood-brain-barrier penetration [63,64,65].

A systematic Cochrane review published by Onwuezobe et al. in 2012 which analyzed antibiotic treatment for symptomatic NTS infection concluded that there was no evidence for the benefit of antibiotics in NTS diarrhea in otherwise healthy people. However, the authors were unable to comment on the antibiotic therapy of very young and very old patients or people with severe and extraintestinal disease as the trials included did not specifically address these subgroups of patients [33].

According to our literature review, adequate antibiotic treatment in vulnerable groups or children already presenting considerably ill with substantially elevated CRP-levels or fever seems to outweigh the questionable risk of a prolongation of fecal NTS excretion. Targeted antibiotic therapy should be performed whenever possible according to antimicrobial activity in vitro susceptibility testing [47,60].

The current recommendation to administer third generation cephalosporins (and ciprofloxacin) for complicated NTS infections should be followed in the light of antimicrobial resistance to these drugs being reported in still low but increasing numbers [66,67,68]. Considered critically important antimicrobials for human medicine, these drugs have been listed in the “Watch”-group of the 2019 WHO AWaRe classification and should be closely monitored in antibiotic surveillance and stewardship programs [69]. As antibiotics do not provide a beneficial effect in the vast majority of uncomplicated *Salmonella* enteritis cases, patients should be selected with scrutiny and restricted use of antibiotics should be a major goal to prevent further development of antibiotic resistance, even more as vaccines against NTS infections are still not available yet [70].

We acknowledge that this review has some limitations. The designs of the included studies were heterogeneous, comprising randomized trials and retrospective cases series, which limits the comparability and the conclusions drawn. Besides that, the quality of the included non-randomized studies was only moderate (*n* = 8) or low (*n* = 1) and the risk of bias for randomized trials was high for three of the five included studies. Another limitation is the heterogeneity of the antibiotic agents administered. Especially antibiotics that had been applied in earlier studies are nowadays no longer common in clinical practice. Furthermore, in the non-randomized studies, the indication for an antibiotic treatment was made depending on the clinical status of the patients (e.g., fever or CRP levels), which is a potential bias for the effect of an antibiotic therapy. In addition, no study explicitly examined other factors, such as coinfections, possibly influencing prolonged excretion of NTS or persistence of symptoms.

## 5. Conclusions

The evidence of protracted NTS excretion after antibiotic treatment in children is mainly derived from older studies using antimicrobials not routinely applied in today’s clinical practice. Recent studies did not observe a prolongation of excretion of NTS and could even show benefits of antibiotic treatment (e.g., ceftriaxone) on the patient’s symptoms in selected cases. This discrepancy between earlier and later observations can be attributed to considerable changes in the antibiotic agents used over time. The restriction of antibiotic treatment for NTS infection, especially in very young children, children with comorbidities, and in those with suspected invasive infection should be critically questioned and the ostensible risk of prolongation of excretion should not lead to a withholding of a treatment with modern antimicrobial agents in these vulnerable subgroups of patients.

## Figures and Tables

**Table 1:** Studies reporting on the duration of excretion and/or the clinical course after antibiotic treatment of NTS infection.

First Author, Year of Publication	Country	Type of Study	Pathogen	Patients (Children) ^1^	Antibiotics	Influence of Antibiotic Treatment on Excretion	Influence of Antibiotic Treatment on Clinical Course
Macdonald, 1954 [31]	Australia	RCS	*S. typhimurium* ^2^	51 (100%)	CHL	no influence	no influence
Dixon, 1965 [32]	England/Wales	RCS	*S. typhimurium*	127 (100%)	NEO, STR, AMP, TET, CHL	prolongation	NA
Rosenstein, 1967 [37]	USA	RCS	NTS	70 (100%)	AMP, NEO, CHL	prolongation	NA
Joint Group, 1970 [41]	England	RCT	NTS	168 (64%) ^4^	NEO	prolongation	no difference in duration of symptoms
Kazemi, 1973 [42]	Canada	RCT	NTS	36 (100%)	SXT, AMP	no influence	no difference in clinical features or duration of symptoms
Kazemi, 1974 [49]	Canada	RCS	NTS	117 (100%)	AMP, PEN, CHL, SXT	prolongation	slight increase in morbidity
Nelson, 1980 [43]	USA	RCT	NTS ^3^	44 (100%)	AMP, AMX	no influence, but more frequent bacteriologic relapse	no influence on symptoms
Stögmann, 1982 [44]	Austria	RCS	NTS	148 (100%)	SXT and/or AMP	no influence	NA
Chiu, 1997 [48]	Taiwan	PCT	NTS	30 (100%)	CRO	shortening	no difference in duration of symptoms
Chiu, 1999 [45]	Taiwan	RCT	NTS	42 (100%)	AZM, CFM	no influence	no difference in duration of symptoms
Barbara, 2000 [23]	Italy	PCS	NTS	1543 (95.1%)	PENs, SXT, CEFs	no influence	higher frequency of persistent symptoms after antibiotic treatment
Lin, 2003 [46]	Taiwan	PCT	NTS	73 (100%)	CRO	no influence	longer duration of fever but rapid defervescence after antibiotic treatment
Tsai, 2011 [13]	Taiwan	RCS	NTS	683 (100%)	AMP, CHL, SXT, CIP, CRO, FLO, CMF, IPM	NA	shorter hospitalization and duration of fever after treatment with CIP or CRO
Ho, 2021 [47]	Taiwan	PCS	NTS	141 (100%)	CRO, SXT, AMP	no influence after appropriate treatment	worse clinical outcome in children with inappropriate antibiotics ^5^

NA: not assessed/not available; NTS: Non-typhoidal *Salmonella* species; ^1^ Patients (absolute number), (children (relative frequency)); ^2^ *S. typhimurium* in 48/51 of cases, 2/51 *S. adelaide*, 1/51 *S. derby*; ^3^ NTS in 43/44 of cases, 1/44 *S. paratyphi*; ^4^ children under the age of 12 years; ^5^ defined as antimicrobial activity in in vitro susceptibility testing. Study type: PCS—prospective case series, RCS—retrospective case series, RCT—randomized controlled trial, PCT—prospective controlled trial. Antibiotics: AMP—ampicillin, AMX—amoxicillin, AZM—azithromycin, CEFs—cephalosporins, CFM—cefixime, CHL—chloramphenicole, CIP—ciprofloxacin, CRO—ceftriaxone, FLO—flomoxef, IPM—imipenem, NEO—neomycin, PEN—penicillin, STR—streptomycin, SXT—trimethoprim-sulfamethoxazole, TET—tetracycline.

**Table 2:** Summary of the findings of this systematic review.

	Findings	Conclusions
Influence of antibiotic treatment on NTS excretion	Prolongation in four studies (all published before 1975)	Most recent studies did not observe a prolongation of NTS excretion.
	Prolongation only after inappropriate antibiotic treatment in one study	
	Shortening in one study	
	No influence in six studies	
Influence of antibiotic treatment on the clinical course of NTS infection	Higher frequency of persistent symptoms in one study	Variable results. Most recent studies did not observe a negative influence of antibiotic treatment on the clinical course.
	Worse clinical outcome only after inappropriate antibiotic treatment in one study	
	Positive effects in two studies	
	No influence in six studies	

## Data Availability

All relevant data are included in this article.

## Supplementary Materials

The following are available online at https://www.mdpi.com/article/10.3390/antibiotics10101187/s1, Table S1: PRISMA 2020 (Preferred Reporting Items for Systematic Review and Meta-Analysis) checklist, Table S2: Definition of the Newcastle-Ottawa Assessment Scale used to the purposes of the present review, Table S3: Quality assessment of non-randomized trials using a modified version of the Newcastle-Ottawa Assessment Scale, Table S4: Quality assessment of randomized controlled trials using the ROB-2 tool.

## References

[1] Stanaway J.D. (2019). The global burden of non-typhoidal salmonella invasive disease: A systematic analysis for the Global Burden of Disease Study 2017. Lancet Infect. Dis..

[2] Havelaar A.H., Kirk M.D., Torgerson P.R., Gibb H.J., Hald T., Lake R.J., Praet N., Bellinger D.C., de Silva N.R., Gargouri N. (2015). World Health Organization Global Estimates and Regional Comparisons of the Burden of Foodborne Disease in 2010. PLoS Med..

[3] Dewey-Mattia D., Manikonda K., Hall A.J., Wise M.E., Crowe S.J. (2018). Surveillance for Foodborne Disease Outbreaks—United States, 2009–2015. MMWR Surveill. Summ..

[4] Butler A.J., Thomas M.K., Pintar K.D.M. (2015). Expert Elicitation as a Means to Attribute 28 Enteric Pathogens to Foodborne, Waterborne, Animal Contact, and Person-to-Person Transmission Routes in Canada. Foodborne Pathog. Dis..

[5] Christidis T., Hurst M., Rudnick W., Pintar K.D.M., Pollari F. (2020). A comparative exposure assessment of foodborne, animal contact and waterborne transmission routes of Salmonella in Canada. Food Control.

[6] Feasey N.A., Archer B.N., Heyderman R.S., Sooka A., Dennis B., Gordon M.A., Keddy K.H. (2010). Typhoid fever and invasive nontyphoid salmonellosis, Malawi and South Africa. Emerg. Infect. Dis..

[7] Kariuki S., Owusu-Dabo E. (2020). Research on Invasive Nontyphoidal Salmonella Disease and Developments Towards Better Understanding of Epidemiology, Management, and Control Strategies. Clin. Infect. Dis..

[8] Scallan E., Hoekstra R.M., Angulo F.J., Tauxe R.V., Widdowson M.A., Roy S.L., Jones J.L., Griffin P.M. (2011). Foodborne illness acquired in the United States—Major pathogens. Emerg. Infect. Dis..

[9] Crump J.A., Sjolund-Karlsson M., Gordon M.A., Parry C.M. (2015). Epidemiology, Clinical Presentation, Laboratory Diagnosis, Antimicrobial Resistance, and Antimicrobial Management of Invasive Salmonella Infections. Clin. Microbiol. Rev..

[10] Takkinsatian P., Silpskulsuk C., Prommalikit O. (2020). Clinical features and antibiotic susceptibility of Salmonella gastroenteritis in children: A ten-year review. Med. J. Malays..

[11] Lounis Y., Hugo J., Demarche M., Seghaye M.C. (2020). Influence of age on clinical presentation, diagnosis delay and outcome in pre-school children with acute appendicitis. BMC Pediatr..

[12] Park S.E., Pak G.D., Aaby P., Adu-Sarkodie Y., Ali M., Aseffa A., Biggs H.M., Bjerregaard-Andersen M., Breiman R.F., Crump J.A. (2016). The Relationship Between Invasive Nontyphoidal Salmonella Disease, Other Bacterial Bloodstream Infections, and Malaria in Sub-Saharan Africa. Clin. Infect. Dis..

[13] Tsai M.H., Huang Y.C., Lin T.Y., Huang Y.L., Kuo C.C., Chiu C.H. (2011). Reappraisal of parenteral antimicrobial therapy for nontyphoidal Salmonella enteric infection in children. Clin. Microbiol. Infect..

[14] Cohen J.I., Bartlett J.A., Corey G.R. (1987). Extra-intestinal manifestations of salmonella infections. Medicine.

[15] Wang J.H., Liu Y.C., Yen M.Y., Wang J.H., Chen Y.S., Wann S.R., Cheng D.L. (1996). Mycotic aneurysm due to non-typhi salmonella: Report of 16 cases. Clin. Infect. Dis..

[16] Zaidi E., Bachur R., Harper M. (1999). Non-typhi Salmonella bacteremia in children. Pediatr Infect. Dis. J..

[17] Mohan A., Munusamy C., Tan Y.C., Muthuvelu S., Hashim R., Chien S.L., Wong M.K., Khairuddin N.A., Podin Y., Lau P.S. (2019). Invasive Salmonella infections among children in Bintulu, Sarawak, Malaysian Borneo: A 6-year retrospective review. BMC Infect. Dis..

[18] Uche I.V., MacLennan C.A., Saul A. (2017). A Systematic Review of the Incidence, Risk Factors and Case Fatality Rates of Invasive Nontyphoidal Salmonella (iNTS) Disease in Africa (1966 to 2014). PLoS Negl. Trop. Dis..

[19] Lehrnbecher T., Laws H.J. (2005). Infectious complications in pediatric cancer patients. Klin. Padiatr..

[20] Huang I.F., Kao C.H., Lee W.Y., Chang M.F., Chen Y.S., Wu K.S., Hu H.H., Hsieh K.S., Chiou C.C. (2012). Clinical manifestations of nontyphoid salmonellosis in children younger than 2 years old—Experiences of a tertiary hospital in southern Taiwan. Pediatr. Neonatol..

[21] Wu H.M., Huang W.Y., Lee M.L., Yang A.D., Chaou K.P., Hsieh L.Y. (2011). Clinical features, acute complications, and outcome of Salmonella meningitis in children under one year of age in Taiwan. BMC Infect. Dis..

[22] Lee W.S., Puthucheary S.D., Parasakthi N. (2000). Extra-intestinal non-typhoidal Salmonella infections in children. Ann. Trop. Paediatr..

[23] Barbara G., Stanghellini V., Berti-Ceroni C., De Giorgio R., Salvioli B., Corradi F., Cremon C., Corinaldesi R. (2000). Role of antibiotic therapy on long-term germ excretion in faeces and digestive symptoms after Salmonella infection. Aliment. Pharmacol. Ther..

[24] Gal-Mor O. (2019). Persistent Infection and Long-Term Carriage of Typhoidal and Nontyphoidal Salmonellae. Clin. Microbiol. Rev..

[25] Marzel A., Desai P.T., Goren A., Schorr Y.I., Nissan I., Porwollik S., Valinsky L., McClelland M., Rahav G., Gal-Mor O. (2016). Persistent Infections by Nontyphoidal Salmonella in Humans: Epidemiology and Genetics. Clin. Infect. Dis..

[26] Buchwald D.S., Blaser M.J. (1984). A review of human salmonellosis: II. Duration of excretion following infection with nontyphi Salmonella. Rev. Infect. Dis..

[27] Devi S., Murray C.J. (1991). Salmonella carriage rate amongst school children—A three year study. Southeast Asian J. Trop. Med. Public Health.

[28] Balasubramanian R., Im J., Lee J.S., Jeon H.J., Mogeni O.D., Kim J.H., Rakotozandrindrainy R., Baker S., Marks F. (2019). The global burden and epidemiology of invasive non-typhoidal Salmonella infections. Hum. Vaccines Immunother..

[29] Mughini-Gras L., Pijnacker R., Duijster J., Heck M., Wit B., Veldman K., Franz E. (2020). Changing epidemiology of invasive non-typhoid Salmonella infection: A nationwide population-based registry study. Clin. Microbiol. Infect..

[30] Marchello C.S., Fiorino F., Pettini E., Crump J.A., Vacc-i N.T.S.C.C. (2021). Incidence of non-typhoidal Salmonella invasive disease: A systematic review and meta-analysis. J. Infect..

[31] Macdonald W.B., Friday F., Mc E.M. (1954). The effect of chloramphenicol in Salmonella enteritis of infancy. Arch. Dis. Child..

[32] Dixon J.M. (1965). Effect of antibiotic treatment on duration of excretion of Salmonella typhimurium by children. Br. Med. J..

[33] Onwuezobe I.A., Oshun P.O., Odigwe C.C. (2012). Antimicrobials for treating symptomatic non-typhoidal Salmonella infection. Cochrane Database Syst. Rev..

[34] Huang I.F., Wagener M.M., Hsieh K.S., Liu Y.C., Wu T.C., Lee W.Y., Chiou C.C. (2004). Nontyphoid salmonellosis in taiwan children: Clinical manifestations, outcome and antibiotic resistance. J. Pediatr. Gastroenterol. Nutr..

[35] Duff N., Steele A.D., Garrett D. (2020). Global Action for Local Impact: The 11th International Conference on Typhoid and Other Invasive Salmonelloses. Clin. Infect. Dis..

[36] Murase T., Yamada M., Muto T., Matsushima A., Yamai S. (2000). Fecal excretion of Salmonella enterica serovar typhimurium following a food-borne outbreak. J. Clin. Microbiol..

[37] Rosenstein B.J. (1967). Salmonellosis in infants and children. J. Pediatr..

[38] Wen S.C., Best E., Nourse C. (2017). Non-typhoidal Salmonella infections in children: Review of literature and recommendations for management. J. Paediatr. Child. Health.

[39] Wells G., Shea B., O’Connell D., Peterson J., Welch V., Losos M., Tugwell P. The Newcastle-Ottawa Scale (NOS) for Assessing the Quality of Nonrandomised Studies in Meta-Analyses. http://www.ohri.ca/programs/clinical_epidemiology/oxford.asp.

[40] Sterne J.A.C., Savovic J., Page M.J., Elbers R.G., Blencowe N.S., Boutron I., Cates C.J., Cheng H.Y., Corbett M.S., Eldridge S.M. (2019). RoB 2: A revised tool for assessing risk of bias in randomised trials. BMJ.

[41] Brown E.H., Laing Brown G., Latham Brown D., Emond R.T.D., Galpine J.F., Jamieson S.R., Lamb S.G., Lambert H.P., McKendrick G.D.W., Medlock J.M. (1970). Effect of neomycin in non-invasive salmonella infections of the gastrointestinal tract. Joint Project by Members of the Association for the Study of Infectious Disease. Lancet.

[42] Kazemi M., Gumpert T.G., Marks M.I. (1973). A controlled trial comparing sulfametboxazole-trimethoprim, ampicillin, and no therapy in the treatment of salmonella gastroenteritis in children. J. Pediatr..

[43] Nelson J.D., Kusmiesz H., Jackson L.H., Woodman E. (1980). Treatment of Salmonella gastroenteritis with ampicillin, amoxicillin, or placebo. Pediatrics.

[44] Stögmann W., Blümel P. (1982). Salmonellosen im Kindersalter—Ein aktuelles Problem (Salmonella enteritis in childhood—A topical problem). Wien. Klin. Wochenschr..

[45] Chiu C.H., Lin T.Y., Ou J.T. (1999). A clinical trial comparing oral azithromycin, cefixime and no antibiotics in the treatment of acute uncomplicated Salmonella enteritis in children. J. Paediatr. Child. Health.

[46] Lin T.Y., Chiu C.H., Lin P.Y., Wang M.H., Su L.H., Lin T.Y. (2003). Short-term ceftriaxone therapy for treatment of severe non-typhoidal Salmonella enterocolitis. Acta Paediatr..

[47] Ho P.Y., Chen W.L., Cheng M.F., Shen Y.T., Hu H.H., Sheu S.K., Huang I.F. (2021). Factors affecting fecal excretion time in pediatric nontyphoid Salmonella infection. Pediatr. Neonatol..

[48] Chiu C.H., Lin T.Y., Ou J.T. (1997). A pilot study of seven days of ceftriaxone therapy for children with Salmonella enterocolitis. Changgeng Yi Xue Za Zhi.

[49] Kazemi M., Gumpert G., Marks M.I. (1974). Clinical spectrum and carrier state of nontyphoidal salmonella infections in infants and children. Can. Med. Assoc. J..

[50] Ruiz M., Rodriguez J.C., Escribano I., Royo G. (2004). Available options in the management of non-typhi Salmonella. Expert Opin. Pharmacother..

[51] Robinson J.L. (2019). Salmonella infections in Canadian children. Paediatr. Child. Health.

[52] Cohen R., Raymond J., Gendrel D. (2017). Antimicrobial treatment of diarrhea/acute gastroenteritis in children. Arch. Pediatr..

[53] Büttcher M., Flieger A., Fruth A., Simon S., Huppertz H.-I., Berner R., Bialek R., Forster J. (2018). Salmonellose. Handbook of the German Society for Pediatric Infectious Diseases—DGPI.

[54] Kimberlin D.W., Barnett E.D., Lynfield R., Sawyer M.H., AAP (2021). Salmonella infections. Red Book—Report of the Committee on Infectious Diseases.

[55] Tack B., Vanaenrode J., Verbakel J.Y., Toelen J., Jacobs J. (2020). Invasive non-typhoidal Salmonella infections in sub-Saharan Africa: A systematic review on antimicrobial resistance and treatment. BMC Med..

[56] Aserkoff B., Bennett J.V. (1969). Effect of antibiotic therapy in acute salmonellosis on the fecal excretion of salmonellae. N. Engl. J. Med..

[57] Pitkäjärvi T., Kujanne E., Sillantaka I., Lumio J. (1996). Norfloxacin and Salmonella excretion in acute gastroenteritis--a 6-month follow-up study. Scand. J. Infect. Dis..

[58] Sanchez C., Garcia-Restoy E., Garau J., Bella F., Freixas N., Simo M., Lite J., Sanchez P., Espejo E., Cobo E. (1993). Ciprofloxacin and trimethoprim-sulfamethoxazole versus placebo in acute uncomplicated Salmonella enteritis: A double-blind trial. J. Infect. Dis..

[59] Smith E.R., Badley B.W. (1971). Treatment of Salmonella enteritis and its effect on the carrier state. Can. Med. Assoc. J..

[60] Shen Y., Huang I., Hu H., Chang M., Sheu S. (2014). Whether Antimicrobial Therapy Affect Fecal Excretion Time In Paediatric Patients Of Nontyphoid Salmonellosis With Different Severity. Arch. Dis. Child..

[61] Yeung C.Y., Lee H.C., Chao Y.N., Chiu N.C., Huang F.Y., Hsieh M.A. (2004). Effect of Antibiotic Therapy on Salmonella Fecal Excretion Time. J. Pediatr. Gastroenterol. Nutr..

[62] Bula-Rudas F.J., Rathore M.H., Maraqa N.F. (2015). Salmonella Infections in Childhood. Adv. Pediatr..

[63] Carlstedt G., Dahl P., Niklasson P.M., Gullberg K., Banck G., Kahlmeter G. (1990). Norfloxacin treatment of salmonellosis does not shorten the carrier stage. Scand. J. Infect. Dis..

[64] Neill M.A., Opal S.M., Heelan J., Giusti R., Cassidy J.E., White R., Mayer K.H. (1991). Failure of ciprofloxacin to eradicate convalescent fecal excretion after acute salmonellosis: Experience during an outbreak in health care workers. Ann. Int. Med..

[65] Kumta N., Roberts J.A., Lipman J., Wong W.T., Joynt G.M., Cotta M.O. (2020). A Systematic Review of Studies Reporting Antibiotic Pharmacokinetic Data in the Cerebrospinal Fluid of Critically Ill Patients with Uninflamed Meninges. Antimicrob. Agents Chemother..

[66] Iwamoto M., Reynolds J., Karp B.E., Tate H., Fedorka-Cray P.J., Plumblee J.R., Hoekstra R.M., Whichard J.M., Mahon B.E. (2017). Ceftriaxone-Resistant Nontyphoidal Salmonella from Humans, Retail Meats, and Food Animals in the United States, 1996–2013. Foodborne Pathog. Dis..

[67] Su L.H., Chiu C.H., Chu C., Ou J.T. (2004). Antimicrobial resistance in nontyphoid Salmonella serotypes: A global challenge. Clin. Infect. Dis..

[68] Kariuki S., Gordon M.A., Feasey N., Parry C.M. (2015). Antimicrobial resistance and management of invasive Salmonella disease. Vaccine.

[69] Sharland M., Pulcini C., Harbarth S., Zeng M., Gandra S., Mathur S., Magrini N., 21st WHO Expert Committee on Selection and Use of Essential Medicines (2018). Classifying antibiotics in the WHO Essential Medicines List for optimal use-be AWaRe. Lancet Infect. Dis..

[70] Baliban S.M., Lu Y.J., Malley R. (2020). Overview of the Nontyphoidal and Paratyphoidal Salmonella Vaccine Pipeline: Current Status and Future Prospects. Clin. Infect. Dis..

