# Supplementary material for: Non-Typhoidal Salmonella Infection in Children: Influence of Antibiotic Therapy on Postconvalescent Excretion and Clinical Course—A Systematic Review
Source: Antibiotics (Basel). 2021 Sep 29;10(10):1187. doi: 10.3390/antibiotics10101187 (PMC8532930; doi:10.3390/antibiotics10101187)
Supplement: Supplementary file 1 [file antibiotics-10-01187-s001.zip › antibiotics-1365301-supplementary.pdf]

Supplementary Materials

Table S1: PRISMA 2020 (Preferred Reporting Items for Systematic Review and Meta-Analysis) checklist.

Section and Topic	Item #	Checklist item	Reported on Page No.
**TITLE**			
Title	1	Identify the report as a systematic review.	1,3
**ABSTRACT**			
Abstract	2	See the PRISMA 2020 for Abstracts checklist.	(see Table below)
**INTRODUCTION**			
Rationale	3	Describe the rationale for the review in the context of existing knowledge.	1,2
Objectives	4	Provide an explicit statement of the objective(s) or question(s) the review addresses.	2
**METHODS**			
Eligibility criteria	5	Specify the inclusion and exclusion criteria for the review and how studies were grouped for the syntheses.	3,4
Information sources	6	Specify all databases, registers, websites, organisations, reference lists and other sources searched or consulted to identify studies. Specify the date when each source was last searched or consulted.	3
Search strategy	7	Present the full search strategies for all databases, registers and websites, including any filters and limits used.	3
Selection process	8	Specify the methods used to decide whether a study met the inclusion criteria of the review, including how many reviewers screened each record and each report retrieved, whether they worked independently, and if applicable, details of automation tools used in the process.	3,4
Data collection process	9	Specify the methods used to collect data from reports, including how many reviewers collected data from each report, whether they worked independently, any processes for obtaining or confirming data from study investigators, and if applicable, details of automation tools used in the process.	3
Data items	10a	List and define all outcomes for which data were sought. Specify whether all results that were compatible with each outcome domain in each study were sought (e.g. for all measures, time points, analyses), and if not, the methods used to decide which results to collect.	3
	10b	List and define all other variables for which data were sought (e.g. participant and intervention characteristics, funding sources). Describe any assumptions made about any missing or unclear information.	3
Study risk of bias assessment	11	Specify the methods used to assess risk of bias in the included studies, including details of the tool(s) used, how many reviewers assessed each study and whether they worked independently, and if applicable, details of automation tools used in the process.	3, Table S2
Effect measures	12	Specify for each outcome the effect measure(s) (e.g. risk ratio, mean difference) used in the synthesis or presentation of results.	Not applicable
Synthesis methods	13a	Describe the processes used to decide which studies were eligible for each synthesis (e.g. tabulating the study intervention characteristics and comparing against the planned groups for each synthesis (item #5)).	3
	13b	Describe any methods required to prepare the data for presentation or synthesis, such as handling of missing summary statistics, or data conversions.	Not applicable
	13c	Describe any methods used to tabulate or visually display results of individual studies and syntheses.	Not applicable
	13d	Describe any methods used to synthesize results and provide a rationale for the choice(s). If meta-analysis was performed, describe the model(s), method(s) to identify the presence and extent of statistical heterogeneity, and software package(s) used.	Not applicable
	13e	Describe any methods used to explore possible causes of heterogeneity among study results (e.g. subgroup analysis, meta-regression).	Not applicable

Section and Topic	Item #	Checklist item	Reported on Page No.
	13f	Describe any sensitivity analyses conducted to assess robustness of the synthesized results.	Not applicable
Reporting bias assessment	14	Describe any methods used to assess risk of bias due to missing results in a synthesis (arising from reporting biases).	3, Table S2
Certainty assessment	15	Describe any methods used to assess certainty (or confidence) in the body of evidence for an outcome.	3
**RESULTS**			
Study selection	16a	Describe the results of the search and selection process, from the number of records identified in the search to the number of studies included in the review, ideally using a flow diagram.	3-7
	16b	Cite studies that might appear to meet the inclusion criteria, but which were excluded, and explain why they were excluded.	3,4
Study characteristics	17	Cite each included study and present its characteristics.	3,7
Risk of bias in studies	18	Present assessments of risk of bias for each included study.	3, Table S3
Results of individual studies	19	For all outcomes, present, for each study: (a) summary statistics for each group (where appropriate) and (b) an effect estimate and its precision (e.g. confidence/credible interval), ideally using structured tables or plots.	3-7
Results of syntheses	20a	For each synthesis, briefly summarise the characteristics and risk of bias among contributing studies.	Not applicable
	20b	Present results of all statistical syntheses conducted. If meta-analysis was done, present for each the summary estimate and its precision (e.g. confidence/credible interval) and measures of statistical heterogeneity. If comparing groups, describe the direction of the effect.	Not applicable
	20c	Present results of all investigations of possible causes of heterogeneity among study results.	Not applicable
	20d	Present results of all sensitivity analyses conducted to assess the robustness of the synthesized results.	Not applicable
Reporting biases	21	Present assessments of risk of bias due to missing results (arising from reporting biases) for each synthesis assessed.	Not applicable
Certainty of evidence	22	Present assessments of certainty (or confidence) in the body of evidence for each outcome assessed.	Not applicable
**DISCUSSION**			
Discussion	23a	Provide a general interpretation of the results in the context of other evidence.	8-10
	23b	Discuss any limitations of the evidence included in the review.	8-10
	23c	Discuss any limitations of the review processes used.	8-10
	23d	Discuss implications of the results for practice, policy, and future research.	8-10
**OTHER INFORMATION**			
Registration and protocol	24a	Provide registration information for the review, including register name and registration number, or state that the review was not registered.	Not applicable
	24b	Indicate where the review protocol can be accessed, or state that a protocol was not prepared.	Not applicable
	24c	Describe and explain any amendments to information provided at registration or in the protocol.	Not applicable
Support	25	Describe sources of financial or non-financial support for the review, and the role of the funders or sponsors in the review.	Not applicable
Competing interests	26	Declare any competing interests of review authors.	10
Availability of data, code and other materials	27	Report which of the following are publicly available and where they can be found: template data collection forms; data extracted from included studies; data used for all analyses; analytic code; any other materials used in the review.	Not applicable

PRISMA 2020 for Abstracts Checklist			
**Section and Topic**	**Item #**	**Checklist item**	**Reported (Yes/No)**
**TITLE**			
Title	1	Identify the report as a systematic review.	Yes
**BACKGROUND**			
Objectives	2	Provide an explicit statement of the main objective(s) or question(s) the review addresses.	Yes
**METHODS**			
Eligibility criteria	3	Specify the inclusion and exclusion criteria for the review.	Yes
Information sources	4	Specify the information sources (e.g. databases, registers) used to identify studies and the date when each was last searched.	Yes
Risk of bias	5	Specify the methods used to assess risk of bias in the included studies.	Yes
Synthesis of results	6	Specify the methods used to present and synthesise results.	Not applicable
**RESULTS**			
Included studies	7	Give the total number of included studies and participants and summarise relevant characteristics of studies.	Yes
Synthesis of results	8	Present results for main outcomes, preferably indicating the number of included studies and participants for each. If meta-analysis was done, report the summary estimate and confidence/credible interval. If comparing groups, indicate the direction of the effect (i.e. which group is favoured).	Yes
**DISCUSSION**			
Limitations of evidence	9	Provide a brief summary of the limitations of the evidence included in the review (e.g. study risk of bias, inconsistency and imprecision).	Yes
Interpretation	10	Provide a general interpretation of the results and important implications.	Yes
**OTHER**			
Funding	11	Specify the primary source of funding for the review.	Not applicable
Registration	12	Provide the register name and registration number.	Not applicable

Table S2: Definition of the Newcastle-Ottawa Assessment Scale used to the purposes of the present review.

**Category**	**Criteria to fulfill**	
**Selection**	Representativeness of the exposed cohort	Children with NTS infection treated with antibiotics
	Selection of the non-exposed cohort	Children with NTS infection without antibiotic treatment
	Ascertainment of exposure	Antibiotic agent, dose, duration of treatment
	Demonstration that outcome of interest was not present at start of study	Positive stool culture for NTS
**Comparability**	Comparability of cohorts based on design or analysis	Two points for randomized trials or, alternatively, if propensity score matching was performed
		One point if other matching methods, involving for example criteria regarding age, severity of illness were applied
**Outcome**	Assessment of outcome	Prospective data collection
	Long enough follow-up	At least 30 days
	Adequacy of follow-up	Complete data for all subjects

Table S3: Quality assessment of non-randomized trials using a modified version of the Newcastle-Ottawa Assessment Scale.

Study	Selection				Comparability	Outcome			Total rating
	Representativeness of the exposed cohort	Selection of the non-exposed cohort	Ascertain-ment of treatment	Demonstration that outcome of interest was not present at start of study	Comparability of cohorts based on design or analysis	Assessment of outcome	Long enough follow-up	Adequate follow-up	Assessment of bias risk
**Dixon, 1965 [32]**	+	+	-	+	-	-	+	+	5 (moderate quality)
**Rosenstein, 1967 [37]**	-[1]	-[1]	-	+	-	-	+	+	3 (low quality)
**Kazemi, 1974 [49]**	+	+	+	+	-	-	+	+	6 (moderate quality)
**Stögmann, 1982 [44]**	+	+	-	+	-	-	+	+	5 (moderate quality)
**Chiu, 1997 [48]**	+	+	+	+	-	+	-	+	6 (moderate quality)
**Barbara, 2000 [23]**	-[2]	-[2]	+	+	-	+	+	+	5 (moderate quality)
**Lin, 2003 [46]**	-[3]	-[3]	+	+	-	+	+	+	5 (moderate quality)
**Tsai, 2011 [13]**	+	+	+	+	-	-	+	+	6 (moderate quality)
**Ho, 2021 [47]**	+	+	-	+	-	+	+	+	6 (moderate quality)

[1]High heterogeneity of included patients; [2]Only patients ≥3 years of age were included; [3]Only patients >6 months of age were included

Table S4: Quality assessment of randomized controlled trials using the ROB-2 tool.

Study	Bias arising from the randomization process	Bias due to deviations from intended interventions	Bias due to missing outcome data	Bias in measurement of the outcome	Bias in selection of the reported result	Overall risk of bias
**Macdonald, 1954 [31]**	Low	Low	Low	Low	Low	Low
**Joint Group, 1970 [41]**	Low	Low	High	Low	Low	High
**Kazemi, 1973 [42]**	Low	High	Low	Low	Low	High
**Nelson, 1980 [43]**	Low	Low	Low	Low	Low	Low
**Chiu, 1999 [45]**	Low	High	High	Low	Low	High
